# Resveratrol Downregulates Granulocyte-Macrophage Colony-Stimulating Factor-Induced Oncostatin M Production through Blocking of PI3K/Akt/NF-κB Signal Cascade in Neutrophil-like Differentiated HL-60 Cells

**DOI:** 10.3390/cimb44020037

**Published:** 2022-01-22

**Authors:** Na-Ra Han, Hi-Joon Park, Phil-Dong Moon

**Affiliations:** 1 College of Korean Medicine, Kyung Hee University, Seoul 02447, Korea; nrhan@khu.ac.kr; 2 Korean Medicine-Based Drug Repositioning Cancer Research Center, College of Korean Medicine, Kyung Hee University, Seoul 02447, Korea; 3 Department of Anatomy & Information Sciences, College of Korean Medicine, Kyung Hee University, Seoul 02447, Korea; acufind@khu.ac.kr; 4 Center for Converging Humanities, Kyung Hee University, Seoul 02447, Korea

**Keywords:** oncostatin M, resveratrol, neutrophil-like differentiated HL-60 cells, PI3K, Akt, NF-κB

## Abstract

Oncostatin M (OSM) is essential in a wide range of inflammatory responses, and most OSM is produced by neutrophils in respiratory diseases. While resveratrol (RES) is regarded as an anti-inflammatory agent in a variety of conditions, the mechanism of OSM inhibition by RES in neutrophils remains to be elucidated. In this study, we investigated whether RES could inhibit OSM production in neutrophil-like differentiated (d)HL-60 cells. The effects of RES were measured by means of an enzyme-linked immunosorbent assay, real-time polymerase chain reaction, and Western blotting. Increases in production and mRNA expression of OSM resulted from the addition of granulocyte-macrophage colony-stimulating factor (GM-CSF) in neutrophil-like dHL-60 cells; however, these increases were downregulated by RES treatment. Exposure to GM-CSF led to elevations of phosphorylation of phosphatidylinositol 3-kinase (PI3K), Akt, and nuclear factor (NF)-kB. Treatment with RES induced downregulation of the phosphorylated levels of PI3K, Akt, and NF-κB in neutrophil-like dHL-60 cells. These results suggest that RES could be applicable to prevent and/or treat inflammatory disorders through blockade of OSM.

## 1. Introduction

Oncostatin M (OSM) is known as a cancer-associated cytokine that is highly expressed in patients with tumors [1,2]. OSM is a member of the interleukin (IL)-6 family cytokines, and is released from a variety of cells, including macrophages, dendritic cells, activated T lymphocytes, monocytes, and neutrophils [3,4,5]. Cytokine OSM plays a role in various pathophysiologic conditions, such as cancer progression, extracellular matrix reconstruction, hemopoiesis, liver regeneration, heart remodeling, and inflammatory reactions [2,6,7,8,9]. OSM plays a role in a wide range of inflammatory responses [2]. Inflammatory reactions in joint disease and hepatic disease involve OSM [2,10]. In addition, OSM is involved in respiratory inflammatory disorders, including allergic rhinitis and asthma [11,12]. Treatment with OSM protein enhanced inflammatory responses in human intestinal stromal cell line CCD-18Co cells [3]. It was reported that OSM stimulation elevates inflammatory reactions in human keratinocyte cell line HaCaT cells [13]. Our previous report also suggested that recombinant OSM treatment upregulates inflammatory cytokine IL-1β production, indicating a contribution of OSM to inflammatory reactions [14]. Pothoven and colleagues [12] suggested that OSM is produced mainly in neutrophils in respiratory inflammatory disorders. However, there are no reports that provide a mechanism for OSM inhibition by resveratrol (RES) in neutrophils. Hence, we examined whether RES inhibits OSM expression in neutrophil-like differentiated (d)HL-60 cells.

It was widely known that phosphatidylinositol 3-kinase (PI3K) is important in regulating a variety of intracellular signaling processes [15]. Akt is a downstream kinase of PI3K, and responsible for inflammatory responses [16]. It is believed that the PI3K/Akt signaling pathway is critical in triggering and amplifying the cytokine system [17]. Lv and colleagues [18] suggested that the PI3K/AKT signal cascade is regarded as a crucial factor in the treatment of various disorders, such as tumorigenesis, cardiovascular problems, and inflammatory reactions. Activated Akt induced the activation of nuclear factor (NF)-κB (a downstream molecule of Akt) [17]. It is known that NF-κB plays an important role as a transcription factor in chronic inflammatory reactions [19]. Su et al. [19] reported that PI3K/Akt/NF-κB signaling pathways are involved in OSM expression in osteoblasts.

**Figure 1:**
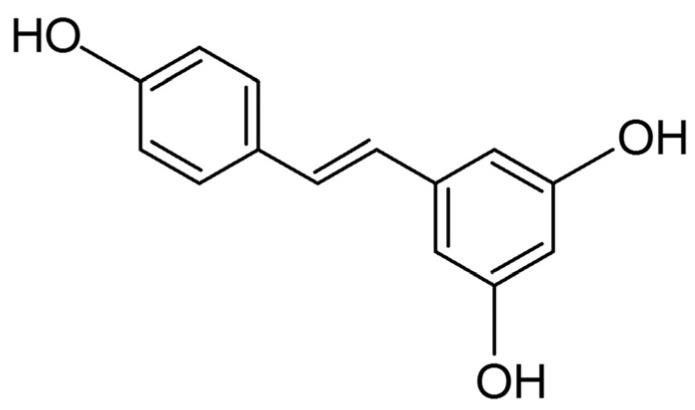
Chemical structure of resveratrol.

Resveratrol (RES, Figure 1) is a well-known dietary polyphenolic compound found in numerous plant species, including peanuts, grapes, mulberry, pines, apples, knotweed, blueberries, and plums [20,21]. RES is beneficial to human health because of its various biological properties, such as anti-cardiovascular, anti-oxidant, anti-obesity, anti-diabetic, anti-inflammatory, anti-viral, neuroprotective, anti-microbial, and anti-cancer effects [21,22,23,24,25]. However, the effect of RES on OSM expression has not been fully clarified. We thus investigated whether RES could inhibit OSM expression in neutrophil-like dHL-60 cells.

## 2. Materials and Methods

### 2.1. Materials 

RES (C_14_H_12_O_3_) was purchased from Sigma-Aldrich Inc. (St. Louis, MO, USA). OSM antibodies and granulocyte-macrophage colony-stimulating factor (GM-CSF) were purchased from R&D Systems (Minneapolis, MN, USA). In Western blotting, phosphorylated (p)-PI3K p85 was purchased from Cell Signaling Technology (Danvers, MA, USA), and the others were purchased from Santa Cruz Biotechnology (Santa Cruz, CA, USA). 

### 2.2. Cells

HL-60 cells were cultured in RPMI 1640 (Gibco BRL, Grand Island, NY, USA) containing 10% (*v*/*v*) heat-inactivated fetal bovine serum (FBS) (Welgene, Daegu, Korea), 100 IU/mL penicillin, and 100 µg/mL streptomycin. To prepare the neutrophilic phenotype dHL-60 cells, HL-60 cells were incubated with 1.3% DMSO for 7 days. A total of 5 ng/mL of recombinant human GM-CSF was used to stimulate the cells, considering the reports of Elbjeirami et al. [26] and Han et al. [14].

### 2.3. Cytotoxicity

dHL-60 cells (1 × 10^5^) were exposed to RES or PBS for 1 h and incubated with 5 ng/mL of GM-CSF for 4 h. The cytotoxicity was examined as previously described [27,28,29,30].

### 2.4. OSM Assay 

dHL-60 cells (5 × 10^5^) were exposed to RES or PBS for 1 h and incubated with 5 ng/mL of GM-CSF for 4 h. OSM levels were measured using an enzyme-linked immunosorbent assay, as previously described [31,32,33,34].

### 2.5. Real-Time Polymerase Chain Reaction (PCR) 

dHL-60 cells (1 × 10^6^) were exposed to RES or PBS for 1 h and incubated with 5 ng/mL of GM-CSF for 1 h. Real-time PCR was conducted as previously described [35,36,37,38].

### 2.6. Western Blotting 

dHL-60 cells (5 × 10^6^) were exposed to RES or PBS for 1 h and incubated with 5 ng/mL of GM-CSF for 15 min (PI3K) or 30 min (Akt) or 1 h (NF-κB). Western blotting was performed, as previously described [39,40,41,42].

### 2.7. Statistical Analysis

One-way ANOVA, followed by the Tukey post hoc test and independent *t*-test, was utilized to analyze the statistically significant differences between the means (IBM SPSS Statistics version 25, Armonk, NY, USA). The statistical significance was set at *p* < 0.05.

## 3. Results

### 3.1. RES Decreases OSM Production in Neutrophil-like dHL-60 Cells 

**Figure 2:**
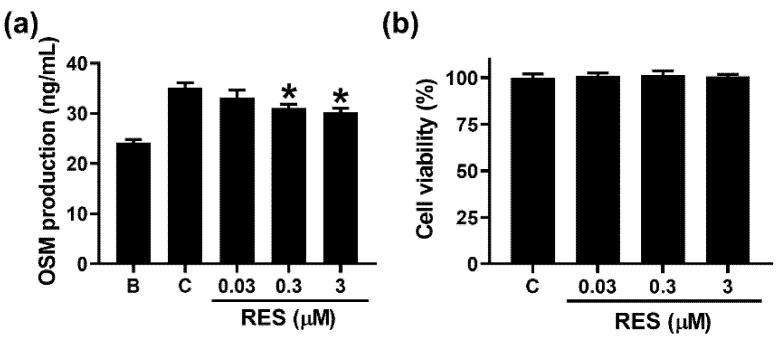
Effects of RES on the production of OSM in neutrophil-like dHL-60 cells. (**a**) dHL-60 cells (5 × 10^5^) were exposed to RES (0.03 to 3 μM) for 1 h, and then stimulated with GM-CSF (5 ng/mL) for 4 h. (**b**) Cytotoxicity was examined using an MTT assay. B, PBS-added and unstimulated cells; C, PBS-added and GM-CSF-stimulated cells. Data are shown as the mean ± SEM of three independent experiments. * *p* < 0.05 vs. the PBS-added, and GM-CSF-stimulated cells.

To investigate whether RES decreases the OSM production in neutrophil-like dHL-60 cells, we added RES into the cells 1 h before GM-CSF treatment. Similar to a previous report [14], increased OSM production resulted from GM-CSF treatment for 4 h (Figure 2a). The addition of RES led to decreased OSM production (Figure 2a). OSM production levels at concentrations of 0.03 to 3 μM were 33.192 ± 1.442, 31.077 ± 0.782, and 30.305 ± 0.752, respectively (Figure 2a). OSM levels in the control and blank groups were 35.148 ± 0.961 and 24.172 ± 0.642, respectively. Cytotoxicity was not shown by the addition of RES (Figure 2b). RES alone did not affect OSM production (Figure S1a).

### 3.2. RES Reducs OSM mRNA Expression in Neutrophil-like dHL-60 Cells

**Figure 3:**
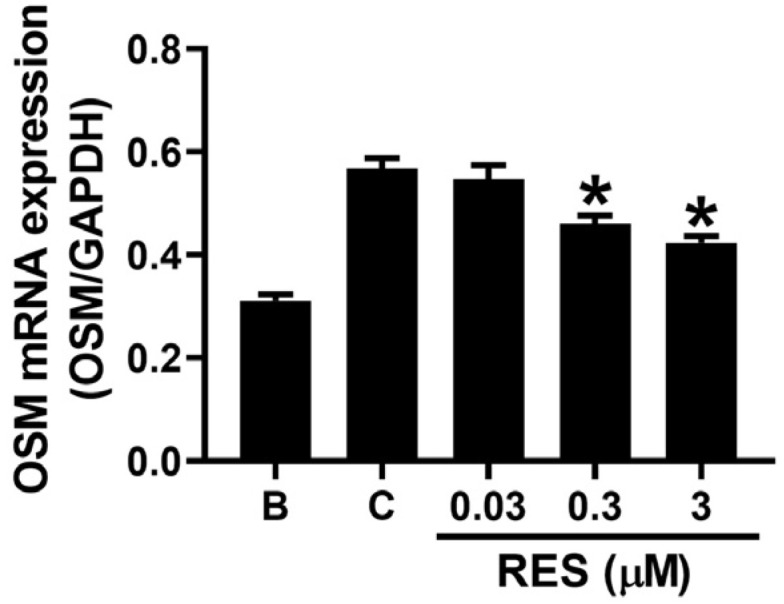
Effects of RES on the mRNA expression of OSM in neutrophil-like dHL-60 cells. dHL-60 cells (1 × 10^6^) were exposed to RES (0.03 to 3 μM) for 1 h, and then stimulated with GM-CSF (5 ng/mL) for 1 h. B, PBS-treated and unstimulated cells; C, PBS-treated and GM-CSF-stimulated cells. B, PBS-added and unstimulated cells; C, PBS-added and GM-CSF-stimulated cells. Data are shown as the mean ± SEM of three independent experiments. * *p* < 0.05 vs. the PBS-added and GM-CSF-stimulated cells.

To evaluate whether RES reduces OSM mRNA expression in neutrophil-like dHL-60 cells, we added RES into the cells 1 h before GM-CSF treatment. Similar to a previous report [14], GM-CSF treatment for 1 h resulted in elevated OSM mRNA expression (Figure 3). The addition of RES led to reduced OSM mRNA expression (Figure 3). The relative levels of OSM mRNA, at concentrations of 0.03 to 3 μM, were 0.547 ± 0.027, 0.461 ± 0.015, and 0.424 ± 0.013, respectively. The levels in the control and blank groups were 0.568 ± 0.021 and 0.311 ± 0.013, respectively. We examined the regulatory effect of 3 μM of RES in the subsequent experiment (Western blotting), because the effect of 3 μM of RES was greater than those of 0.03 and 0.3 μM. RES alone did not affect OSM mRNA expression (Figure S1b).

### 3.3. RES Downregulates Phosphorylation of PI3K in Neutrophil-like dHL-60 Cells 

**Figure 4:**
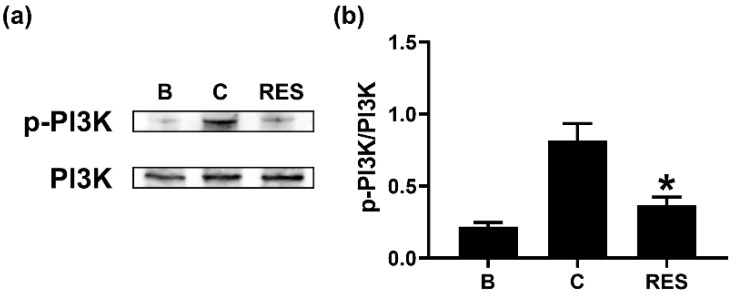
Effects of RES on the phosphorylation of PI3K in neutrophil-like dHL-60 cells. (**a**) dHL-60 cells (5 × 10^6^) were exposed to RES (3 μM) for 1h, and then stimulated with GM-CSF (5 ng/mL) for 15 min. (**b**) The protein levels were quantitated by densitometry. B, PBS-added and unstimulated cells; C, PBS-added and GM-CSF-stimulated cells; RES, RES-added and GM-CSF-stimulated cells. Data are shown as the mean ± SEM of three independent experiments. * *p* < 0.05 vs. the PBS-added and GM-CSF-stimulated cells.

To understand the regulatory mechanism of OSM reduction by RES, we exposed neutrophil-like dHL-60 cells to RES (3 μM) for 1 h. Similar to a previous report [14], GM-CSF treatment for 15 min induced upregulated PI3K phosphorylation (Figure 4). However, the exposure to RES resulted in downregulation of PI3K phosphorylation (Figure 4). RES alone did not affect PI3K phosphorylation (Figure S2a,b).

### 3.4. RES Inhibits Phosphorylation of Akt in Neutrophil-like dHL-60 Cells 

**Figure 5:**
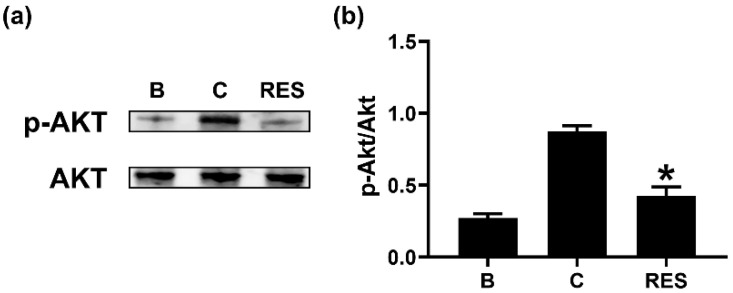
Effects of RES on the phosphorylation of Akt in neutrophil-like dHL-60 cells. (**a**) dHL-60 cells (5 × 10^6^) were exposed to RES (3 μM) for 1h, and then stimulated with GM-CSF (5 ng/mL) for 30 min. (**b**) The protein levels were quantitated by densitometry. B, PBS-added and unstimulated cells; C, PBS-added and GM-CSF-stimulated cells; RES, RES-added and GM-CSF-stimulated cells. Data are shown as the mean ± SEM of three independent experiments. * *p* < 0.05 vs. the PBS-added and GM-CSF-stimulated cells.

To examine the regulatory mechanism of OSM reduction by RES, we exposed neutrophil-like dHL-60 cells to RES (3 μM) for 1 h. Similar to a previous report [14], GM-CSF treatment for 30 min induced elevated Akt phosphorylation (Figure 5). However, the exposure to RES resulted in decreased phosphorylation of Akt (Figure 5). RES alone did not affect Akt phosphorylation (Figure S2c,d).

### 3.5. RES Decreases Phosphorylation of NF-κB in Neutrophil-like dHL-60 Cells 

**Figure 6:**
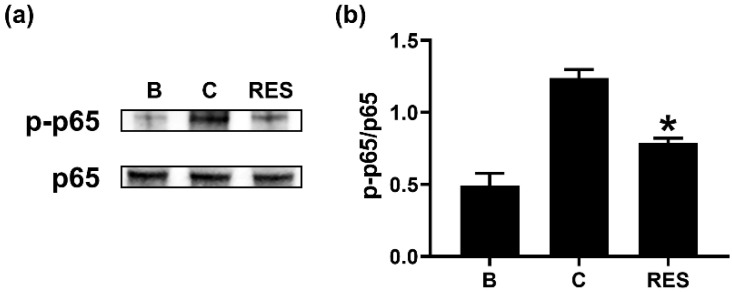
Effects of RES on the phosphorylation of NF-kB in neutrophil-like dHL-60 cells. (**a**) dHL-60 cells (5 × 10^6^) were exposed to RES (3 μM) for 1h, and then stimulated with GM-CSF (5 ng/mL) for 1 h. (**b**) The protein levels were quantitated by densitometry. B, PBS-added and unstimulated cells; C, PBS-added and GM-CSF-stimulated cells; RES, RES-added and GM-CSF-stimulated cells. Data are shown as the mean ± SEM of three independent experiments. * *p* < 0.05 vs. the PBS-added and GM-CSF-stimulated cells.

To understand the regulatory mechanism of OSM reduction by RES, we exposed neutrophil-like dHL-60 cells to RES (3 μM) for 1 h. Similar to a previous report [14], GM-CSF treatment for 1 h led to increased NF-κB phosphorylation (Figure 6). However, a decrease in NF-κB phosphorylation resulted from the exposure to RES (Figure 6). RES alone did not affect NF-κB phosphorylation (Figure S2e,f).

## 4. Discussion

Numerous studies have reported that increased OSM levels are detected in inflammatory diseases, including chronic rhinosinusitis and asthma [12,43,44]. Ma et al. [45] suggested that GM-CSF stimulation induces elevated OSM mRNA expression. Moreover, many studies reported that elevation of OSM resulted from stimulation by GM-CSF in human neutrophils [12,26,46,47]. Similar to our previous report [14], the results of the present study demonstrated that exposure of neutrophil-like dHL-60 cells to GM-CSF results in increased OSM production and mRNA expression (Figure 2a and Figure 3). The increases in OSM production and mRNA expression were attenuated by addition of RES (Figure 2a and Figure 3). Treatment with OSM protein in the nasal cavity led to the infiltration of inflammatory cells and upregulation of inflammatory cytokines and chemokines in a murine model [48]. Modur and colleagues [49] suggested that skin inflammation is increased by hypodermic injection of OSM protein in mice. It was reported [50] that lung inflammation resulted from hyperexpression of OSM in a murine model. High levels of OSM mRNA and protein were exhibited in patients with asthma, whereas no OSM was shown in control subjects [44]. Furthermore, an OSM-deficiency and neutralizing antibody treatment decreased colon inflammation [3]. Thus, we presuppose that RES might be advantageous for use in preventing and/or treating inflammatory diseases through blockade of OSM.

It is widely known that the PI3K/AKT signal pathway plays a pivotal role in inflammatory reactions [15,16,17,18]. NF-κB is a well-known transcription factor of inflammatory responses [19]. It was reported that OSM production is mediated by PI3K/Akt/NF-κB signal pathway in osteoblasts [19]. Our previous report also confirmed a dependency of the PI3K/Akt/NF-κB signal pathway on OSM production in neutrophil-like dHL-60 cells [25]. Treatment with PI3K inhibitor decreased the mRNA expression and protein levels of various inflammatory cytokines, such as IL-1β, IL-6, and tumor necrosis factor (TNF)-α, in nucleus pulposus cells [51]. In addition, blockade of PI3K/Akt signal pathway resulted in reduction of osteoarthritis in mice [52]. Administration of well-known PI3K inhibitors, including wortmannin, LY-294002, and IC87114, suppressed airway hyperresponsiveness and inflammation in a murine model of asthma [53,54]. Furthermore, treatment with an Akt inhibitor (deguelin) downregulated airway inflammation in asthmatic mice [55]. NF-κB inhibition also attenuated airway inflammation and hyperresponsiveness in ovalbumin-induced asthma model [55,56]. Our results showed that RES treatment induced decreases in phosphorylation of PI3K, Akt, and NF-κB (Figure 4, Figure 5 and Figure 6). Thus, we presume that decrease of OSM by RES might be at least partly controlled by PI3K/Akt/NF-κB signaling pathway in neutrophil-like dHL-60 cells.

Cardiovascular and cerebrovascular disorders, together with cancers, constitute the most important causes of death in Europe, the USA, and most Asian countries. It was suggested that RES exerts beneficial effects on cardiovascular diseases [57]. Thaung Zaw et al. [58] reported that RES enhances cerebrovascular function in postmenopausal women. Hence, we assume that RES may be helpful for many people to prevent and/or treat inflammatory diseases, as well as cardiovascular and cerebrovascular disorders, in Europe, USA, and most Asian countries. Therefore, RES could reduce duplication of medication in inflammatory disease patients with cardiovascular and cerebrovascular disorders. 

Lastly, no toxic effect was shown in rats that were administered 300 mg/kg of RES daily for 4 weeks [59]. Here, we utilized 3 μM of RES (approximately 0.684 mg/kg). Hence, we could assume that RES may not be toxic to humans at concentration of 3 μM.

## 5. Conclusions

**Figure 7:**
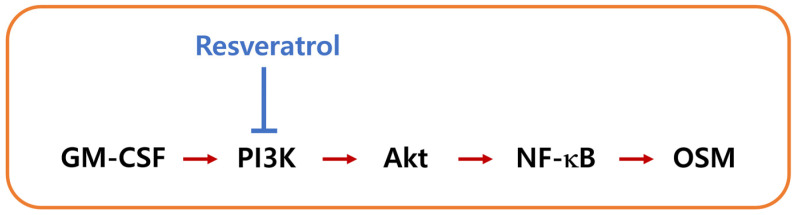
A schematic diagram of the proposed inhibition of OSM by RES.

In conclusion, we showed that RES repressed OSM production via downregulation of PI3K/Akt/NF-κB signal cascade in neutrophil-like dHL-60 cells (Figure 7). The results of the present study suggest that RES may be a useful drug target for inflammatory disorders treatment.

## Data Availability

Data is contained within the article.

## Supplementary Materials

The following are available online at https://www.mdpi.com/article/10.3390/cimb44020037/s1. Figure S1: Effects of RES on the production and mRNA expression of OSM in neutrophil-like dHL-60 cells. Figure S2: Effects of RES on the phosphorylation of PI3K, Akt, and NF-κB in neutrophil-like dHL-60 cells.
