# Supplementary material for: Resveratrol Downregulates Granulocyte-Macrophage Colony-Stimulating Factor-Induced Oncostatin M Production through Blocking of PI3K/Akt/NF-κB Signal Cascade in Neutrophil-like Differentiated HL-60 Cells
Source: Curr Issues Mol Biol. 2022 Jan 22;44(2):541–9. doi: 10.3390/cimb44020037 (PMC8928961; doi:10.3390/cimb44020037)
Supplement: Supplementary file 1 [file cimb-44-00037-s001.zip › cimb-1550759-SI.pdf]

Supplementary

Resveratrol Downregulates Granulocyte-Macrophage Colony-Stimulating Factor-Induced Oncostatin M Production through Blocking of PI3K/Akt/NF-κB Signal Cascade in Neutrophil-like Differentiated HL-60 Cells

Na-Ra Han [1,2], Hi-Joon Park [3] and Phil-Dong Moon [4,*]

[1] College of Korean Medicine, Kyung Hee University, 26, Kyungheedae-ro, Dongdaemun-gu, Seoul, 02447, Republic of Korea; nrhan@khu.ac.kr

[2] Korean Medicine-Based Drug Repositioning Cancer Research Center, College of Korean Medicine, Kyung Hee University, 26, Kyungheedae-ro, Dongdaemun-gu, Seoul, 02447, Republic of Korea

[3] Department of Anatomy & Information Sciences, College of Korean Medicine, Kyung Hee University, 26, Kyungheedae-ro, Dongdaemun-gu, Seoul, 02447, Republic of Korea; acufind@khu.ac.kr

[4] Center for Converging Humanities, Kyung Hee University, 26, Kyungheedae-ro, Dongdaemun-gu, Seoul, 02447, Republic of Korea; pdmoon@khu.ac.kr

* Correspondence: pdmoon@khu.ac.kr; Tel.: +82-2-961-0897

Results

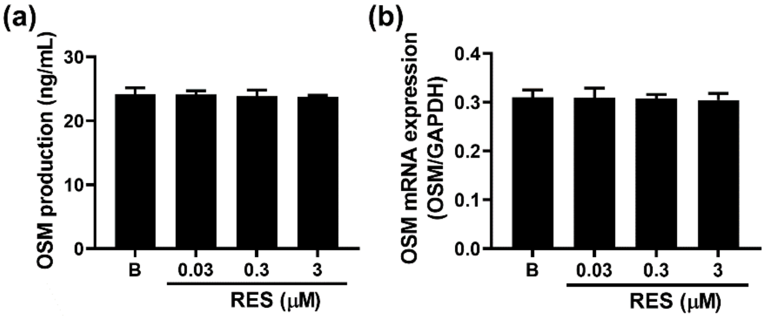

Figure S1. Effects of RES on the production and mRNA expression of OSM in neutrophil-like dHL-60 cells. (**a**) dHL-60 cells (5 × 10^5) were exposed to RES (0.03 to 3 μM) for 1 h, and then incubated without GM-CSF stimulation for 4 h. (**b**) dHL-60 cells (1 × 10^6) were exposed to RES (0.03 to 3 μM) for 1 h, and then incubated without GM-CSF stimulation for 1 h. B, PBS-added, and unstimulated cells. Data are shown as the mean ± SEM of three independent experiments.

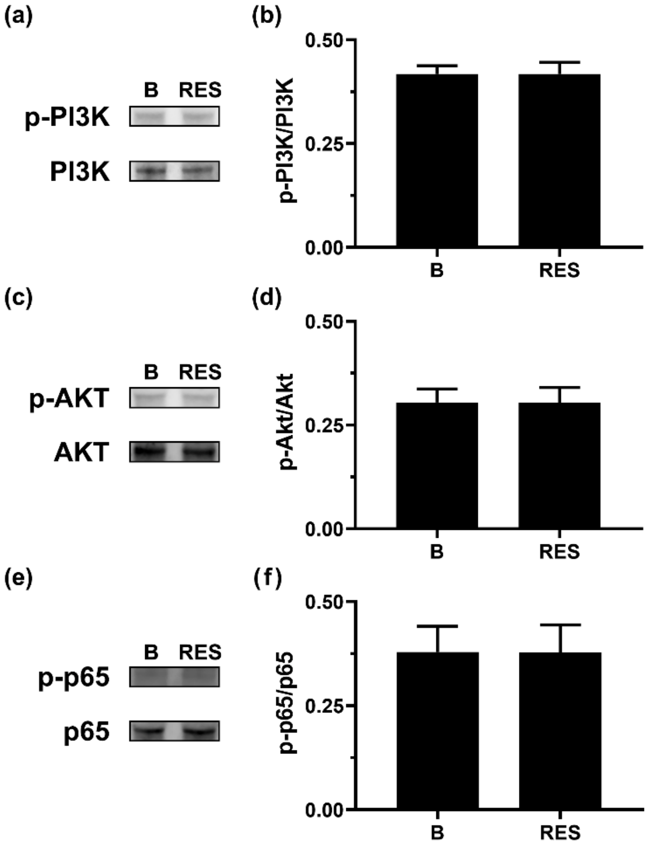

Figure S2. Effects of RES on the phosphorylation of PI3K, Akt, and NF-κB in neutrophil-like dHL-60 cells. (**a**,**c**,**e**) dHL-60 cells (5×10^6) were exposed to RES (3 μM) for 1h, and then incubated without GM-CSF stimulation for 15 min (PI3K), 30 min (Akt), and 60 min (NF-κB). (**b**,**d**,**f**) The protein levels were quantitated by densitometry. B, PBS-added, and unstimulated cells; RES, RES-added, and unstimulated cells. Data are shown as the mean ± SEM of three independent experiments.

Materials and Methods

Cytotoxicity

The dHL-60 cells (1×10^5) were seeded in 24-well plate and pretreated with RES or PBS for 1 h, and then stimulated with GM-CSF for 4 h. The cells were incubated with 3-(4,5-dimethylthiazol-2-yl)-2,5-diphenyltetrazolium bromide (MTT, Sigma-Aldrich Co.) solution at 37 °C for 4 h. Next, we added 1 mL of dimethyl sulfoxide to dissolve the MTT formazan, and transferred 100 μL of supernatant into a new 96-well microplate. A microplate reader (540 nm, Versa Max, Molecular Devices, Sunnyvale, CA, USA) was used to measure the absorbance of formazan dissolved in DMSO [1-4].

OSM Assay

OSM levels were assessed by means of an enzyme-linked immunosorbent assay, as previously described [5-8]. The capture antibody (R&D system Inc., Minneapolis, MN, USA) was pre-coated in a 96-well plate. Phosphate-buffered saline (PBS) containing 10% FBS was added to block the plate for 2 h. After washing the plate by means of PBS containing Tween 20 (PBST), cell supernatants were added into the plate for 2 h. After washing the plate with PBST, the plate was treated with biotinylated detection antibody (R&D system Inc.) for 2 h and then incubated with avidin-conjugated to horseradish peroxidase (Sigma-Aldrich Co.) for 30 min. Absorbance by TMB substrate (BD Pharmingen, San Jose, CA, USA) was measured by a microplate reader (405 nm, Versa Max).

Real-Time PCR

The dHL-60 cells (1 × 10^6) were seeded in 6-well plate and pretreated with RES or PBS for 1 h, and then stimulated with GM-CSF for 1 h. The harvested cells were used to isolate total RNA by means of an RNA extraction reagent (iNtRON, Seongnam, Republic of Korea), as previously described [9-12]. The first-strand cDNA from total RNA was synthesized with cDNA synthesis reagents (Bioneer, Daejeon, Republic of Korea). The following designed primers were used for the real time PCR (Applied Biosystems, Foster City, CA, USA) by using Power SYBR® Green Master Mix (Applied Biosystems): *OSM*: 5′-GCTCACACAGAGGACGCTG-3′, 5′-GGAGCACGCGGTACTCTTTC-3′; *GAPDH*: 5′-TCGACAGTCAGCCGCATCTTCTTT-3′, 5′-ACCAAATCCGTTGACTCCGACCTT-3′. The relative expression of mRNA for OSM was normalized by GAPDH and measured by using $2^{-\Delta\Delta Ct}$ method.

Western Blotting

The dHL-60 cells (5 × 10^6) were seeded in 60 mm dish and pretreated with RES or PBS for 1 h, and then stimulated with GM-CSF for 15 min (PI3K) or 30 min (Akt) or 60 min (NF-κB). Western blot analysis was conducted, as previously described [13-16]. An ice-cold cell lysis buffer (Sigma-Aldrich Co.) was used to lyse the harvested cells. Cell extracts were prepared with sampling buffer (Laemmli's 2×, ELPISBIOTECH. INC., Daejeon, Republic of Korea) and heated at 95 °C for 5 min. Proteins were subjected to electrophoresis using 10% - 15% gel containing sodium dodecyl sulfate and transferred to nitrocellulose membranes (Amersham™, Chicago IL, USA). PBST containing 5% bovine serum albumin (Sigma-Aldrich Co.) was used to block the membranes afterwards relevant primary antibodies (phosphorylated (p)-PI3K, Cell Signaling Technology, Danvers, MA, USA; PI3K, p-Akt, Akt, p-p65, p65, and GAPDH, Santa Cruz Biotechnology, Santa Cruz, CA, USA) were used. Peroxidase-conjugated secondary antibodies (Santa Cruz Biotechnology) were added for incubation of the membranes for 1 h at room temperature after washing with PBST. Specific bands were detected by an enhanced chemiluminescence solution (DoGenBio Co., Seoul, Republic of Korea). Band intensities were calculated with ImageJ program (National health institute, Bethesda, MD, USA).

References

1. Moon, P.D.; Lee, J.S.; Kim, H.Y.; Han, N.R.; Kang, I.; Kim, H.M.; Jeong, H.J. Heat-treated Lactobacillus plantarum increases the immune responses through activation of natural killer cells and macrophages on in vivo and in vitro models. *J. Med. Microbiol.* **2019**, *68*, 467-474. https://doi.org/10.1099/jmm.0.000938
2. Alghamdi, Y.S.; Saleh, O.M.; Alqadri, N.; Mashraqi, M.M.; Bahattab, O.; Awad, N.S. Effect of Ducrosia flabellifolia and Savignya parviflora Extracts on Inhibition of Human Colon and Prostate Cancer Cell Lines. *Curr. Issues Mol. Biol.* **2021**, *43*, 1518-1528. https://doi.org/10.3390/cimb43030107
3. Insuan, O.; Janchai, P.; Thongchuai, B.; Chaiwongsa, R.; Khamchun, S.; Saoin, S.; Insuan, W.; Pothacharoen, P.; Apiwatanapiwat, W.; Boondaeng, A.; Vaithanomsat, P. Anti-Inflammatory Effect of Pineapple Rhizome Bromelain through Downregulation of the NF-B- and MAPKs-Signaling Pathways in Lipopolysaccharide (LPS)-Stimulated RAW264.7 Cells. *Curr. Issues Mol. Biol.* **2021**, *43*, 93-106. https://doi.org/10.3390/cimb43010008
4. Han, N.R.; Ko, S.G.; Moon, P.D.; Park, H.J. (2021). Chloroquine attenuates thymic stromal lymphopoietin production via suppressing caspase-1 signaling in mast cells. *Biomed. Pharmacother.* **2021**, *141*, 111835. https://doi.org/10.1016/j.biopha.2021.111835
5. Moon, P.D.; Han, N.R.; Kim, H.M.; Jeong, H.J. High-Fat Diet Exacerbates Dermatitis through Up-Regulation of TSLP. *J. Invest. Dermatol.* **2019**, *139*, 1198-1201. https://doi.org/10.1016/j.jid.2018.11.003
6. Han, N.R.; Ko, S.G.; Moon, P.D.; Park, H.J. Ginsenoside Rg3 attenuates skin disorders via down-regulation of MDM2/HIF1α signaling pathway. *J. Ginseng Res.* **2021**, *45*, 610–616. https://doi.org/10.1016/j.jgr.2021.06.008
7. Han, N.R.; Kim, H.J.; Lee, J.S.; Kim, H.Y.; Moon, P.D.; Kim, H.M.; Jeong, H.J. (2021). The immune-enhancing effect of anthocyanin-fucoidan nanocomplex in RAW264.7 macrophages and cyclophosphamide-induced immunosuppressed mice. *J. Food Biochem.* **2021**, *45*, e13631. https://doi.org/10.1111/jfbc.13631
8. Moon, P.D.; Han, N.R.; Lee, J.S.; Kim, H.M.; Jeong, H.J. (2021). p-coumaric acid, an active ingredient of Panax ginseng, ameliolates atopic dermatitis-like skin lesions through inhibition of thymic stromal lymphopoietin in mice. *J. Ginseng Res.* **2021**, *45*, 176–182. https://doi.org/10.1016/j.jgr.2020.06.004

9. Han, N.R.; Kim, H.Y.; Kang, S.; Kim, M.H.; Yoon, K.W.; Moon, P.D.; Kim, H.M.; Jeong, H.J. Chrysophanol, an anthraquinone from AST2017-01, possesses the anti-proliferative effect through increasing p53 protein levels in human mast cells. *Inflamm. Res*. **2019**, *68*, 569-579. https://doi.org/10.1007/s00011-019-01239-7
10. Han, N.R.; Moon, P.D.; Kim, H.M.; Jeong, H.J. TSLP Exacerbates Septic Inflammation via Murine Double Minute 2 (MDM2) Signaling Pathway. *J. Clin. Med*. **2019**, *8*, 1350. https://doi.org/10.3390/jcm8091350
11. Alamir, A.H.; Patil, S. Allicin Could Potentially Alleviate Oral Cancer Pain by Inhibiting "Pain Mediators" TNF-alpha, IL-8, and Endothelin. *Curr. Issues Mol. Biol*. **2021**, *43*, 187-196. https://doi.org/10.3390/cimb43010016
12. Kang, M.H.; Jang, G.Y.; Ji, Y.-J.; Lee, J.H.; Choi, S.J.; Hyun, T.K.; Kim, H.D. Antioxidant and Anti-Melanogenic Activities of Heat-Treated Licorice (Wongam, Glycyrrhiza glabra × G. uralensis) Extract. *Curr. Issues Mol. Biol*. **2021**, *43*, 1171-1187. https://doi.org/10.3390/cimb43020083
13. Moon, P.D.; Han, N.R.; Lee, J.S.; Kim, H.M.; Jeong, H.J. Ursolic acid downregulates thymic stromal lymphopoietin through the blockade of intracellular calcium/caspase-1/NF-κB signaling cascade in HMC-1 cells. *Int. J. Mol. Med*. **2019**, *43*, 2252-2258. https://doi.org/10.3892/ijmm.2019.4144
14. Moon, P.D.; Han, N.R.; Lee, J.S.; Hong, S.; Yoo, M.S.; Kim, H.J.; Kim, J.H.; Kang, S.; Jee, H.W.; Kim, H.M.; et al. Use of Physcion to Improve Atopic Dermatitis-Like Skin Lesions through Blocking of Thymic Stromal Lymphopoietin. *Molecules* **2019**, *24*, 1484. https://doi.org/10.3390/molecules24081484
15. Han, N.R.; Han, S.J.; Moon, P.D.; Hong, S.; Kim, H.; Li, Y.H.; Kim, H.M.; Jeong, H.J. Effect of dexamethasone injection into Zusanli (ST 36) acupoint on ovalbumin-induced allergic rhinitis. *J. Tradit. Chin. Med*. **2019**, *39*, 307-314.
16. Iannucci, J.; Sen, A.; Grammas, P. Isoform-Specific Effects of Apolipoprotein E on Markers of Inflammation and Toxicity in Brain Glia and Neuronal Cells In Vitro. *Curr. Issues Mol. Biol*. **2021**, *43*, 215-225. https://doi.org/10.3390/cimb43010018
